# CT features of electronic-cigarette or vaping-associated lung injury (EVALI); our experience during the recent outbreak

**DOI:** 10.1259/bjrcr.20200027

**Published:** 2020-03-26

**Authors:** Francis Girvin, David Naidich

**Affiliations:** 1 NYU Langone Health, New York, United States

## Abstract

As an emerging clinical syndrome, our knowledge of the clinical, pathologic and radiologic features of electronic-cigarette or vaping-associated lung injury is evolving. CT appearances are described in six cases imaged at our institution (NYU Langone Health, New York) in the cluster of 2019.

## Introduction

CT patterns are described in six cases that satisfied the Center of Disease Control (CDC) case definition of confirmed or probable electronic-cigarette or vaping-associated lung injury (EVALI) imaged at our institution between September and December 2019. The CDC description of imaging findings is vague, limited to “pulmonary infiltrates, such as opacities on plain film, or groundglass opacities on chest CT”. The purpose of this case review series is to add to the evolving radiologic literature of case reports, correspondence and review articles that provide more specific descriptions of the typical CT patterns encountered.

## Cases (see Table 1 for summary of imaging findings)

**Table 1.** Summary of CT findings

Case	M/F	Age yrs	Groundglass	Consolidation	Septal thickening	Zonal distribution	Centrilobular opacities	Peribronchovascular opacities	Peripheral opacities	Subpleural or lobular sparing	Effusions	Lymphadenopathy
1	F	35	Yes	Yes	Yes	Lower	Yes	Yes	Yes	Yes	Trace	Yes
2	F	19	Yes	Yes	Yes	Lower	Yes	Yes	Yes	Yes	No	Yes
3	M	20	Yes	Yes	Yes	Lower	Yes	Yes	Yes	Yes	No	Yes
4	F	45	Yes	Yes	Yes	Lower	Yes	Yes	Yes	Yes	Trace	Yes
5	M	19	Yes	No	Yes	None	No	Yes	No	No	No	Yes
6	M	46	Yes	Yes	No	Lower	Yes	Yes	Yes	Yes	No	Yes

### Case 1

**Figure 1.**
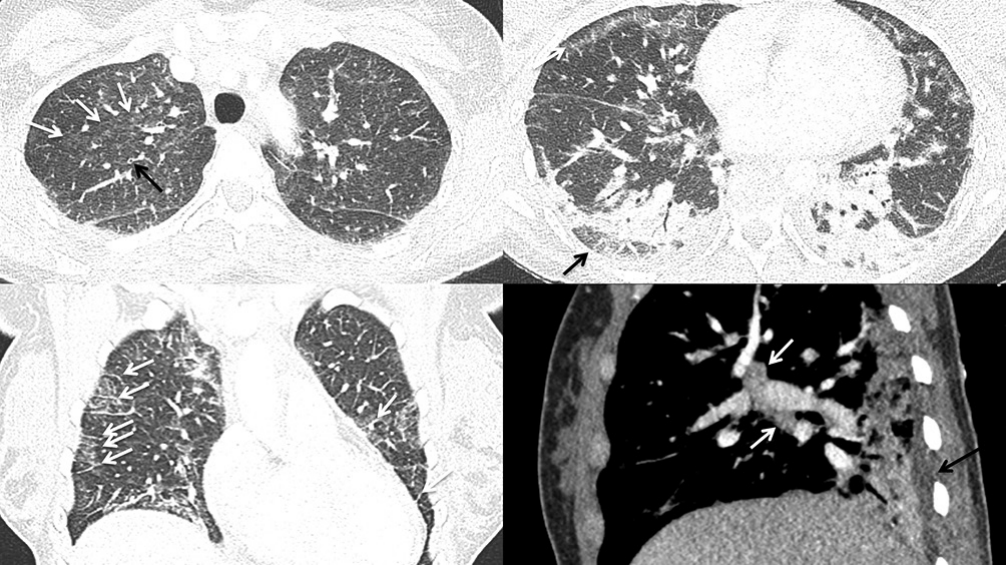
A (top left). Axial HRCT image showing centrilobular groundglass opacities (white arrows) and bronchial wall thickening (black arrow) in the upper lobes B (top right). Axial HRCT showing showing groundglass opacities with subpleural sparing (white arrow) in the right middle lobe and lingula and peribronchovascular and peripheral consolidation in the lower lobes with subpleural sparing (black arrow) C (bottom left). Coronal CT image showing groundglass opacities and septal thickening (white arrows) D (bottom right). Sagital CT image showing mild hilar lymphadenopathy (white arrows) and trace pleural effusions (black arrow). HRCT, high-resolution CT

A 35-year-old female with a history of daily THC (tetrahydrocannabinol) vaping presented with 6 days of dyspnea, cough, chest pain, low grade fever, loss of appetite, nausea, loose stools, and fatigue. Notable laboratory indices revealed a mildly elevated white blood cell count (WBC) of 13.5. Pro-calcitonin and infectious screen were negative. Chest CT (Figure 1) demonstrated lower lung predominant airspace opacities (a combination of groundglass and consolidation) with subpleural sparing, mild centrilobular groundglass opacities in the upper lobes, mild septal thickening, bronchial wall thickening, trace pleural effusions, and mild mediastinal and hilar lymphadenopathy measuring up to 1.5 cm.

### Case 2

**Figure 2.**
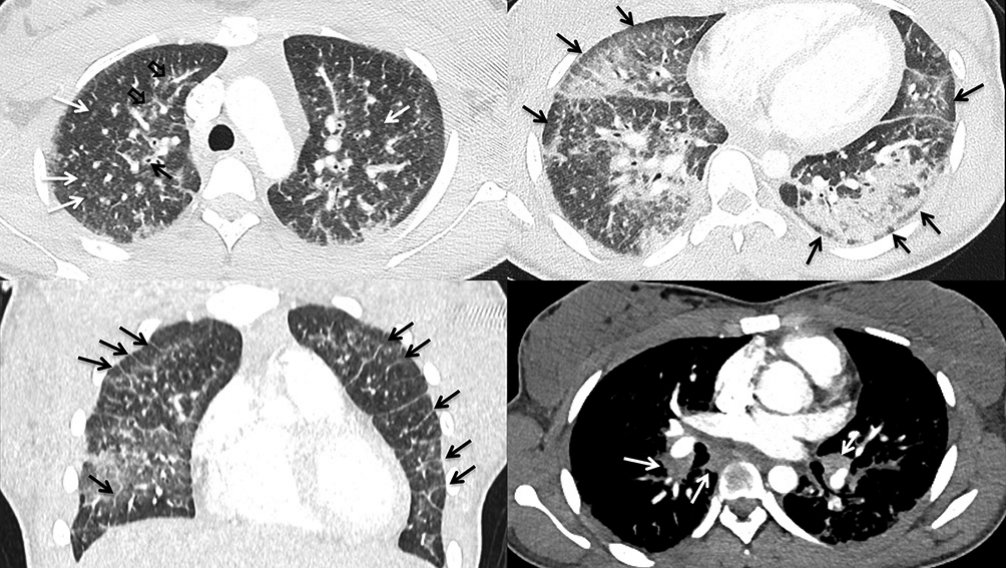
A (top left). Axial HRCT image showing both centriolbular (white arrows) and peribronchovascular (black open arrows) and severe bronchial wall thickening in the upper lobes B (top right). Axial HRCT image showing lower lung predominant peribronchovascular groundglas and consolidation with subpleural sparing (black arrows) C (bottom left). Coronal CT image showing bilateral groundglass opacities with septal thickening (black arrows) D (bottom right). Axial CT image showing mild subcarinal and bihilar lymphadenopathy. HRCT, high-resolution CT.

A 19-year-old female with a history of daily THC vaping for 2 years presented with 5 days of dry cough, substernal chest pain, fever, chills, nausea, post-tussive vomiting, loss of appetite and diarrhea. Notable laboratory indices included a mildy elevated WBC of 13 and significantly elevated erythrocyte sedimentation rate (ESR) of >130 and C-reactive protein (CRP) of 208. Bronchoalveolar lavage (BAL) demonstrated Gram +ve cocci in pairs, and abundant histiocytes, lymphocytes, neutrophils and many lipid-laden macrophages. Chest CT (Figure 2) demonstrated lower lung predominant airspace opacities (a combination of consolidation and groundglass), areas of lobular and subpleural sparing, extensive diffuse bronchial wall thickening, septal thickening, and mild mediastinal and hilar lymphadenopathy measuring up to 1.4 cm. Despite the isolation of Gram +ve cocci on BAL, the case satisfied the CDC criteria for “probable” EVALI as per the clinical team’s assessment.

### Case 3

**Figure 3.**
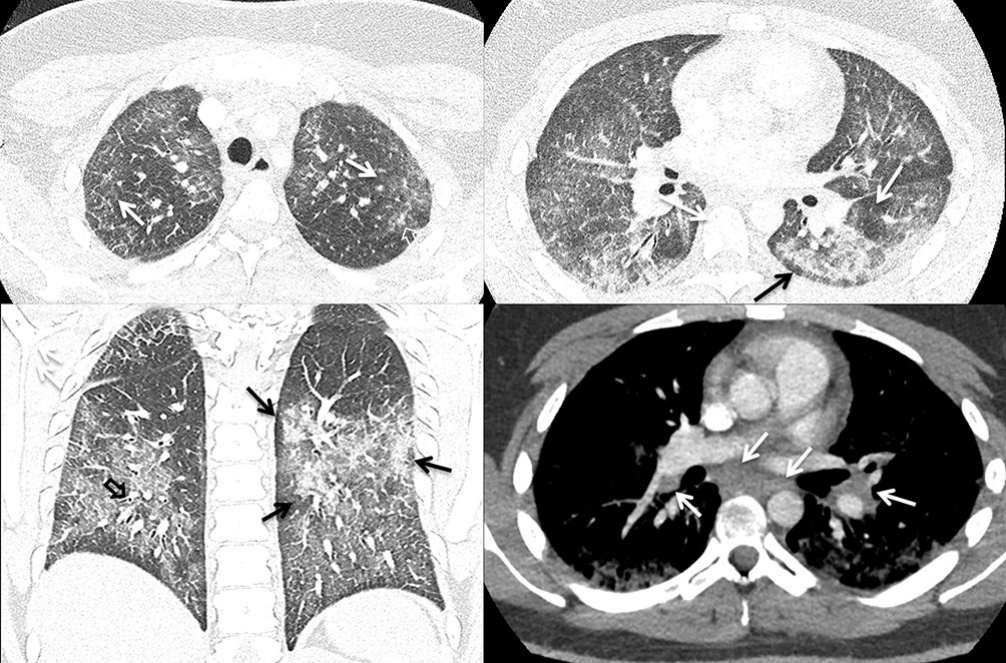
A (top left). Axial HRCT image showing both centrilobular (white arrows) and peribronchovascular (open white arrow) in the apices B (top right). Axial HRCT image showing peribronchovascular groundglass and consolidation with lobular (white arrows) and subpleural (black arrow) sparing C (bottom left). Coronal CT image showing peribronchovascular groundglass and consolidation (black arrows), bronchial wall thickening (black open arrow), and mild septal thickening (white arrows) D (bottom right). Axial CT image showing mild subcarinal and hilar lymphadenopathy (white arrows). HRCT, high-resolution CT.

A 20-year-old male with a 2 year history of daily vaping of THC and nicotine products presented with 1 week of cough with clear sputum, dyspnea, fever, fatigue and myalgias. Notable laboratory indices included an elevated WBC of 27 and transaminitis. Infectious screening was negative. Chest CT (Figure 3) demonstrated lower lung predominant groundglass opacities with areas of lobular and subpleural sparing, mild diffuse bronchial wall thickening, mild septal thickening, and mild mediastinal and hilar lymphadenopathy measuring up to 1.4 cm.

### Case 4

**Figure 4.**
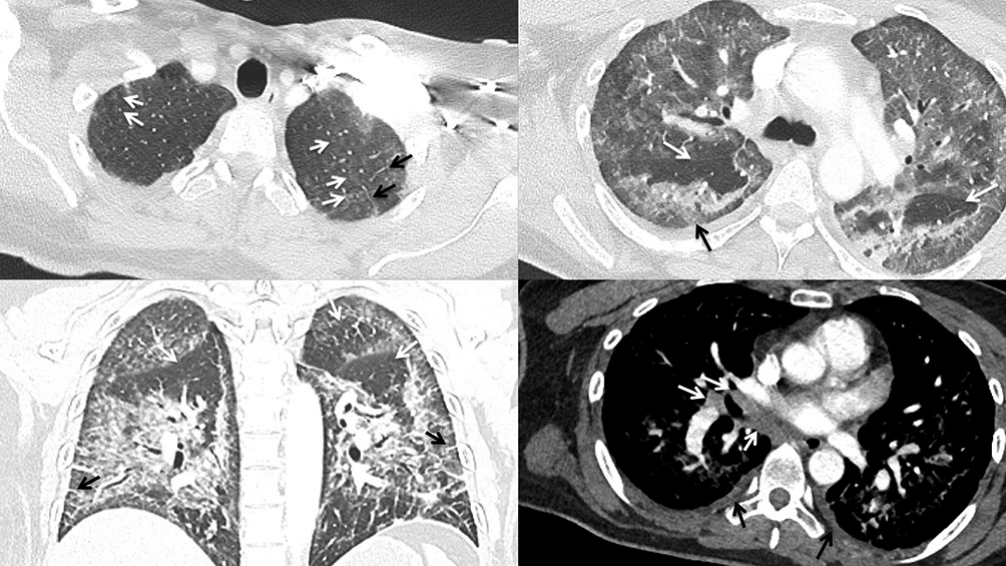
A (top left). Axial HRCT image showing mild centrilobular groundglass opacities in the both apices (white arrows) and scant interlobular septal thickening at the left apex (black arrows) B (top right). Axial HRCT image showing bilateral geographic airspace opacities with lobular (white arrows) and subpleural (black arrow) sparing C (bottom left). Coronal CT image showing bilateral lower lobe predominant airspace opacities with lobular sparing (white arrows) and mild septal thickening (black arrows) D (bottom right). Axial CT image showing mild subcarinal and right hilar lymphadenopathy (white arrows). HRCT, high-resolution CT.

A 45-year-old female with a prior history of smoking marijuana for chronic pain control presented after recently switching to vaping THC with several days of dyspnea and fever. Notable laboratory indices included an elevated WBC of 15 and transaminitis, and infectious work-up was negative. Chest CT (Figure 4) demonstrated lower lung predominant groundglass opacities with prominent lobular and subpleural sparing, mild centrilobular groundglass nodules in the upper lobes, mild septal thickening, trace pleural effusions, and mild mediastinal and hilar lymphadenopathy measuring up to 1.1 cm.

### Case 5

**Figure 5.**
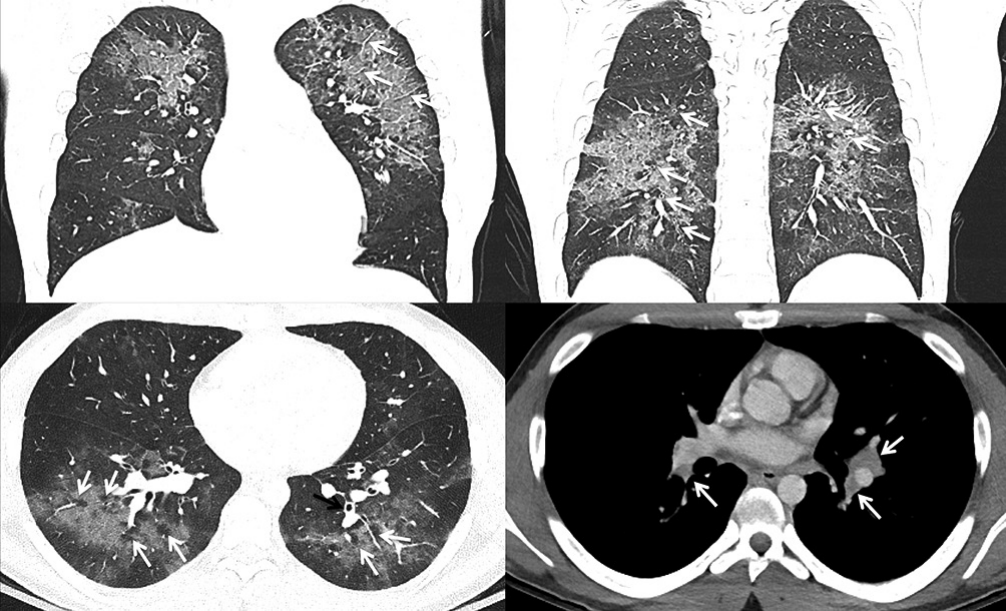
A (top left). Coronal CT image showing bilateral upper lobe peribronchovascular groundglass opacities, with mild septal thickening in the left upper lobe (white arrows) B (top right). Coronal CT image showing bilateral lower lobe peribronchovascular groundglass opacities, with bronchial wall thickening (white arrows) C (bottom left). Axial HRCT image showing bilateral lower lobe peribronchovascular groundglass opacities with unusual small areas of sparing in the immediate peribronchovascular space (white arrows) and bronchial wall thickening (black arrow) D (bottom right). Axial CT image showing mild bilateral hilar lymphadenopathy (white arrows). HRCT, high-resolution CT.

An 18-year-old male with history of daily THC vaping presented with 3 days of cough, dyspnea and chest pain. Notable laboratory indices included an elevated WBC of 21. Infectious work-up was negative. Chest CT (Figure 5) demonstrated a geographic pattern of predominantly peribronchovascular groundglass opacities without zonal predominance, with small areas of sparing in the immediate peribronchovascular lung, and scant septal thickening. Mild mediastinal and hilar lymphadenopathy was also present, measuring up to 1.2 cm.

### Case 6

**Figure 6.**
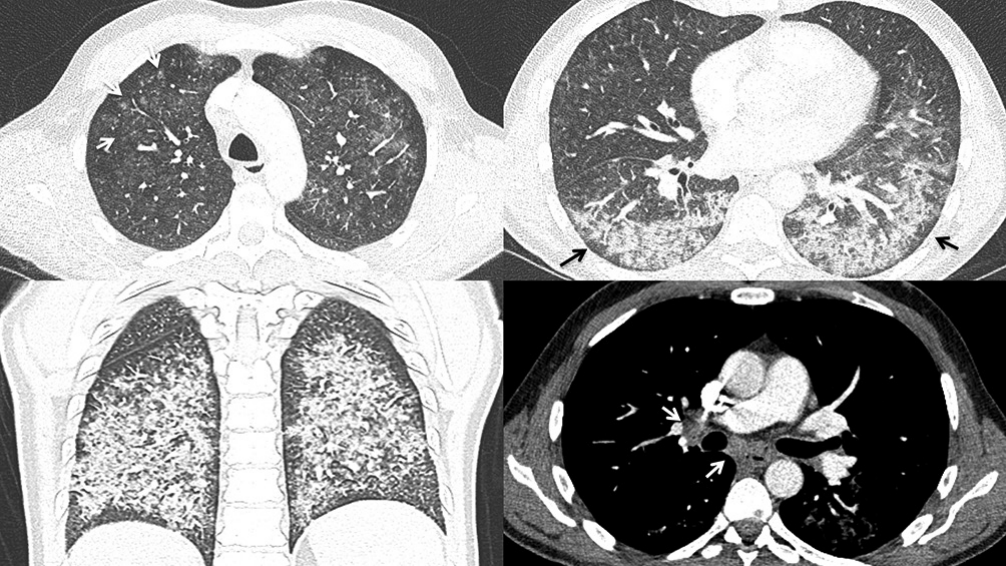
A (top left). Axial HRCT image showing profuse centrilobular groundglass nodules in the upper lobes (white arrows) B (top right). Axial HRCT image showing peribronchovascular airspace opacities in the lower lobes with subpleural sparing (black arrows) C (bottom left). Coronal CT image showing lower lung predominant peribronchovascular airspace opacities D (bottom right). Axial CT image showing mild subcarinal and right hilar lymphadenopathy (white arrows). HRCT, high-resolution CT.

A 46-year-old male with a history of regular THC vaping presented with 2 weeks of dry cough, malaise, fever, nausea, vomiting and diarrhea. Notable laboratory indices included a borderline elevated WBC of 12 and an elevated CRP of 110 and ESR of 120. Infectious work-up was negative. Chest CT (Figure 6) demonstrated profuse centrilobular groundglass opacities in the upper lungs and coalescent peribronchovascular groundglass and consolidation in the lower lungs with lobular and subpleural sparing. Mild mediastinal and hilar lymphadenopathy was also present, measuring up to 1.3 cm. See table for summary of findings.

## Discussion

The United States outbreak of EVALI began in June 2019, reaching a peak in September. As of January 7, 2020, a total of 2602 cases of EVALI and 57 EVALI-related deaths have been reported to CDC across the United States. The CDC case definition is broad, requiring use of an e-cigarette or dabbing (the inhalation of vaporized cannabis oil or resin) within 90 days before symptom onset, pulmonary “infiltrates” on imaging, negative infectious work-up, and no plausible alternative diagnosis.^1^ Cases can also be considered “probable” rather than “confirmed” when infectious screening is incomplete, or if infection is present but felt unlikely to account for the degree of lung injury. Onset of symptoms tends to be subacute. Respiratory symptoms are typically the dominant presenting complaint, however, gastrointestinal and constitutional symptoms are also common.

All of the patients in this series had a history of THC vaping, and overwhelming evidence supports that use of THC-containing products in particular poses a specific risk for lung injury, with the majority (82%) of hospitalized patients reporting a history of THC use.^1^

All patients in our series had peribronchovascular airspace opacities, the majority with areas of either lobular or subpleural sparing—a frequently recognized pattern in organizing pneumonia.^2^ While the changes affected all lobes in every case, a lower zonal predominance was present in all but one case that demonstrated equal involvement in all zones (Case 6). Bronchial wall thickening and centrilobular groundglass supportive of airway inflammation was also present in the majority, consistent with histopathological descriptions of bronchiolitis in EVALI.^3^ The majority also showed mild septal thickening, however, airspace disease was overwhelmingly the predominant finding in all cases. Trace effusions were less frequent findings in two cases. All had mild, presumably reactive mediastinal and hilar lymphadenopathy. Although lipid laden macrophages have been described in the BAL fluid in approximately 50% of cases,^4,5^ and were present in the single case from this series who underwent BAL, macroscopic fat on CT was not identified in any of our cases, and has not been described in other published reports, correspondence or review articles pertaining to this outbreak.

Layden et al briefly summarized chest CT findings in 48 of 53 patients of EVALI who underwent CT, with 100% of those cases having an “abnormal chest CT”, characteristically showing bilateral groundglass opacities, sometimes with subpleural sparing.^4^ Henry et al^6^ and Kligerman et al^7^ have provided more specific radiologic descriptions of the imaging findings in EVALI, and consistent with our own experience also described appearances compatible with organizing pneumonia and diffuse alveolar damage, with peripheral or lobular sparing, commonly with upper lobe predominant centrilobular nodules, and septal thickening.

As expected from the imaging patterns encountered, pathologic descriptions in EVALI are compatible with acute lung injury—Butt et al described lung biopsy results from 17 patients, with variable patterns of acute lung injury, including acute fibrinous pneumonitis, diffuse alveolar damage, and/or organizing pneumonia, usually bronchiolocentric and accompanied by bronchiolitis.^3^

In important work towards determining potential etiologic factors accounting for the recent outbreak, Blount et al^8^ analyzed BAL fluid and isolated vitamin E acetate in 48 (94%) of 51 cases of EVALI, in contrast to no detectable levels in fluid from 99 healthy volunteers (including 18 asymptomatic e-cigarette users). Postulated toxic effects of vitamin E acetate on pulmonary function have included a decrease in surfactant surface tension, or the formation of potentially lung-irritant ketene after heating vitamin E acetate within the electronic delivery device. Alternatively, vitamin E acetate may be a marker for alternative toxic agents as yet to be determined.

Although the appropriate treatment strategy for EVALI remains to be validated, Layden et al reported clinical improvement in the majority of patients treated with systemic steroids.^4^ All of our patients received steroid therapy, and all had symptomatic improvement and radiographic regression of lung opacities on follow up plain films prior to discharge.

## Conclusion

Similar to published correspondence and review articles to date, our experience of the CT appearances of EVALI during the nationwide outbreak of late 2019 revealed the presence of lower lung predominant airspace opacities (groundglass and/or consolidation), with lobular and/or subpleural sparing, diffuse bronchial wall thickening, mild interlobular septal thickening, and mild likely reactive lymphadenopathy as the most typical radiologic findings. Trace effusions were less frequent occasional features. The presence of these findings, especially in younger patients with acute or subacute respiratory complaints should prompt specific enquiries regarding vaping exposure—particularly THC, if not already volunteered or solicited at the time of presentation. Further descriptions regarding the radiologic patterns of EVALI are to be expected within the literature, given the scale of the recent outbreak and should add to the evolving knowledge base of this recently defined clinical entity.

## Learning points

Peribronchovascular airspace opacities with lobular or subpleural sparing and mild septal thickening are compatible with EVALI in patients with a history of exposure and exclusion of other plausible alternative etiologies.
